# A novel BK channel-targeted peptide suppresses sound evoked activity in the mouse inferior colliculus

**DOI:** 10.1038/srep42433

**Published:** 2017-02-14

**Authors:** L. L. Scott, E. J. Brecht, A. Philpo, S. Iyer, N. S. Wu, S. J. Mihic, R. W. Aldrich, J. Pierce, J. P. Walton

**Affiliations:** 1 Waggoner Center for Alcohol and Addiction Research, University of Texas at Austin, Austin, USA; 2 Department of Chemical & Biomedical Engineering, University of South Florida, Tampa, USA; 3 Center for Learning and Memory, University of Texas at Austin, Austin, USA.; 4 Department of Communication Sciences & Disorders, University of South Florida, Tampa, Florida, United States of America; 5 Global Center for Hearing & Speech Research, University of South Florida, Tampa, Florida, United States of America

## Abstract

Large conductance calcium-activated (BK) channels are broadly expressed in neurons and muscle where they modulate cellular activity. Decades of research support an interest in pharmaceutical applications for modulating BK channel function. Here we report a novel BK channel-targeted peptide with functional activity *in vitro* and *in vivo*. This 9-amino acid peptide, LS3, has a unique action, suppressing channel gating rather than blocking the pore of heterologously expressed human BK channels. With an IC_50_ in the high picomolar range, the apparent affinity is higher than known high affinity BK channel toxins. LS3 suppresses locomotor activity via a BK channel-specific mechanism in wild-type or BK channel-humanized *Caenorhabditis elegans*. Topical application on the dural surface of the auditory midbrain in mouse suppresses sound evoked neural activity, similar to a well-characterized pore blocker of the BK channel. Moreover, this novel ion channel-targeted peptide rapidly crosses the BBB after systemic delivery to modulate auditory processing. Thus, a potent BK channel peptide modulator is open to neurological applications, such as preventing audiogenic seizures that originate in the auditory midbrain.

The large conductance calcium-activated potassium (BK) channel is a promising pharmacological target. Widely expressed in human tissue where it is gated by voltage and intracellular calcium, the BK channel regulates smooth muscle tone, endocrine secretion and neuronal excitability^1^. Despite broad expression, global knockout of the constitutive pore-forming α subunit is not lethal in mice^2^. In *Caenorhabditis elegans* and *Drosophila*, null mutations in the highly conserved invertebrate BKα channel reduces acute ethanol intoxication and tolerance^3^,^4^.

Restriction of BK channel function has therapeutic value in human disease. Human tumor growth and metastasis is supported by higher BK channel expression and curtailed by BK channel blockers^5^,^6^. In the CNS, a BK channel gain-of-function mutation is associated with an increased risk for epilepsy in humans and mice^7^,^8^, and blocking BK channel function suppresses seizure activity *in vivo* and *in vitro*^9^,^10^. BK channels are expressed throughout the auditory system where they maintain high frequency firing^11^,^12^,^13^. Thus, suppression of BK channel function in the inferior colliculus (IC) may restrict audiogenic seizures, which originate in this region^14^,^15^,^16^. A reduction in high frequency firing in the IC could also reduce tinnitus or “ringing in the ears.” Evidence from animal models of tinnitus suggests that a focal loss of output from the cochlea, common in many cases of tinnitus, in turn suppresses inhibitory drive in the central auditory system resulting in hyperexcitability^17^. This neuronal hyperexcitability is evident in both the cochlear nucleus and at the level of the IC^18^,^19^.

A number of peptides alter BK channel function. Peptide scorpio- or conotoxins block the pore with low nanomolar affinity and high specificity but have relatively complex structures restricting large-scale synthesis^20^. Small, endogenous peptides or peptide fragments modulate BK channel function, but mainly act with lower affinity^21^,^22^,^23^,^24^. For example, fragments of a BK channel auxiliary subunit (β2) inactivate currents in the micromolar range^23^,^24^. There are currently no BK channel-directed peptides for CNS pharmacological applications, having high affinity and specificity, blood-brain barrier (BBB) permeability and amenability to large-scale synthesis.

Here we report that a novel 9-amino acid peptide, LS3, is a high affinity BK channel closer suitable for CNS applications *in vivo*. Extracellular application of LS3 reduced the probability of human BK channel opening with a mid-picomolar IC_50_. LS3 displays BK channel-specific activity in a *C. elegans* behavioral assay. Topical application on the dural surface of the mouse auditory midbrain suppressed sound evoked neural activity in the inferior colliculus, similar to the known BK channel pore blocker, paxilline. Together these data show that LS3 significantly alters mammalian BK channel gating and crosses the blood-brain barrier (BBB) to suppress neural activity *in vivo*. Through suppression of neural activity in the IC, LS3 could provide therapeutic anti-convulsant or tinnitus-reduction activity.

## Results

**Figure 1:**
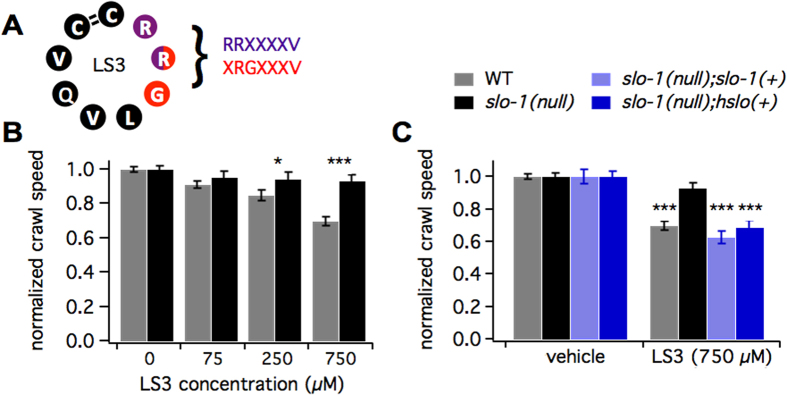
LS3 alters BK channel function in wild type or humanized *C. elegans*. (**A**) Schematic of LS3 shows the sequence, 1–9 disulfide bridge and two enriched motifs (purple and red residues, enriched ~4000 and ~3000 fold, respectively, during the selection process). (**B**) Bar graph of crawl speed normalized to vehicle-treated controls shows that LS3 reduced crawl speed for wild-type (gray bars) but not *slo*-*1* null (black bars) worms in a concentration dependent manner (WT vs. *slo*-*1*(*null*) at 250 and 750 μM: p < 0.05 and p < 0.001, N = 139–372, Two-way ANOVA with Dunn’s post-hoc correction). (**C**) Bar graph of crawl speed normalized to vehicle-treated controls shows that reduction in crawl speed by 750 μM LS3 was rescued on the *slo*-*1* null background with extrachromosomal expression of either the worm (*slo*-*1*(+)) or the human (*hslo*(+)) BK channel gene (vs. *slo*-*1*(*null*): p < 0.001 for both, N = 78–162, Two-way ANOVA with Dunn’s post-hoc correction). Worms were bathed in LS3 or vehicle for 30 min prior to tracking crawl speed.

Here we characterize a novel BK channel-directed peptide, LS3. LS3 was selected as a potential BK channel modulator using phagemid display. We employed a cyclic, 9-amino acid scaffold with scalable synthesis and resistance to proteolytic degradation^25^,^26^. Phage expressing randomized peptide sequences that bound to the ZERO isoform of the human BKα channel were enriched. One such sequence was CRRGLVQVC, containing two motifs, **RR**XXXXV and X**RG**XXXV, which were enriched ~4000 and ~3000 fold, respectively (Fig. 1A). This sequence was synthesized as LS3 (CRRGLVQVC with a 1–9 disulfide bridge, Fig. 1A, Supp. Fig. 1).

To determine whether LS3 modulates BK channel function *in vivo* with minimal off-target effects, we employed a *Caenorhabditis elegans* behavioral assay. Animals were preincuabted in either NGM or peptide prior to tracking crawl speed on unseeded plates. Brief peptide application in liquid was chosen to avoid potential catabolic by-products of incubation on metabolically active *E. coli*. Many gene products modulate *C. elegans* neuromuscular circuit activity^27^, including the highly conserved BK channel ortholog, SLO-1^28^,^29^. By testing strains carrying a gene’s WT or null allele, crawl behavior probes for specificity. LS3 slowed locomotion in a concentration-dependent manner (Fig. 1B, Supp. Fig. 1A–C). Pre-incubating worms in a puddle of 250 or 750 μM LS3 for 30 minutes reduced crawl speed to ~85% and ~70% of controls, respectively (p < 0.001). By contrast, the *slo*-*1* null mutant did not significantly slow in response to LS3 (Fig. 1B, Supp. Fig. 2A–C). The LS3-induced slowing response was rescued in the *slo*-*1*(*null*) background by transgenic expression of the wild-type *slo*-*1* gene (Fig. 1C, Supp. Fig. 2D). Because the human BKα channel (HSLO) was used to select LS3, we hypothesized that LS3 would also modulate HSLO function. Consistent with this prediction, transgenic expression of *hslo* in the *slo*-*1*(*null*) background rescued the LS3-induced slowing response (Fig. 1C, Supp. Fig. 2D). Together these data indicate that LS3 alters *C. elegans* locomotor behavior by specific modulation of worm or human BK channel function within an *in vivo* context. As *C. elegans* homologues have been identified for 60–80% of human genes including many highly conserved potassium channels^30^,^31^,^32^,^33^,^34^, the specificity of action for the BK channel found in *C. elegans* likely carries over to mammals. To provide further support for specificity of LS3 for the BK channel, LS3 was screened for binding to a comprehensive panel of mammalian CNS-based proteins using a competition binding assay (NIMH-PDSP). A high concentration of LS3 (10 microM) caused substantially less than 50% inhibition of radioligand binding in all cases (Supp. Table 1). These data suggest that LS3 does not significantly inhibit ligand binding to an array of CNS targets (Ki > 10 microM).

**Figure 2:**
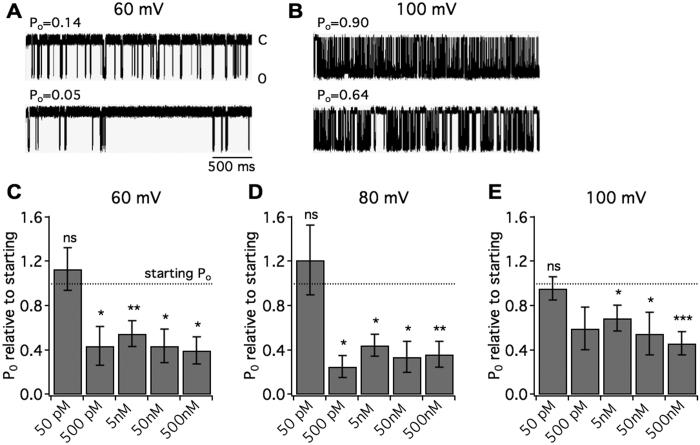
LS3 alters the gating of human BK channels. Human BKα channels (ZERO isoform) expressed in HEK293 cells were recorded in inside-out patches. Peptide was applied by diffusion to the extracellular side. Intracellular calcium held at 750 nM. Each trace is 3 seconds long. (**A**,**B**) Single channel traces show 500 nM LS3 reduces the probability of opening at 60 mV (**A**) and 100 mV (**B**). (**C**–**E**) Bar graphs displaying the post-peptide P_o_ relative to the starting P_o_ across LS3 concentrations. At 500 pM through 500 nM LS3 reduced the P_o_ at each holding potential (pre vs. post: *p < 0.05, **p < 0.01, ***p < 0.001, N = 6–12, planned Student’s t-tests). 50 pM LS3 did not significantly alter the P_o_.

The potency of a compound can be difficult to extrapolate from pharmaco-behavioral analyses in *C. elegans* because high doses are required to cross the worm cuticle. The brief duration (30 minutes) and mode of treatment allowing minimal ingestion (swimming in liquid) further entail high doses^35^. Additionally, the *C. elegans* behavioral assay does not determine the type of activity at the BK channel as both gain- and loss-of-function mutations slow locomotion^3^. To gain a meaningful measure of LS3 potency and molecular action, we performed electrophysiological recordings of human BK channels. LS3 was applied to the extracellular side of inside-out patches containing human BKα channels (ZERO isoform) expressed heterologously in HEK293 cells. Intracellular calcium was held at 750 nM. At high picomolar to nanomolar concentrations, LS3 reduced the probability of channel opening (P_o_) by ~60% at 60 mV (Fig. 2A,B). In contrast, single channel conductance remained stable (pre peptide: 282 ± 4.62 pS; post 500 nM LS3: 287 ± 5.12 pS, measured at 60 mV). LS3 reduced P_o_ at each holding potential (60–100 mV) and from 500 pM to 500 nM (Fig. 2C–E), but did not significantly alter P_o_ at 50 pM at any voltage. Similarly, 500 nM LS3 reduced P_o_ at 40 mV (post P_o_ relative to pre: 0.54 ± 0.17, p < 0.05). These data indicate that LS3 has an IC_50_ in the mid-picomolar range.

**Figure 3:**
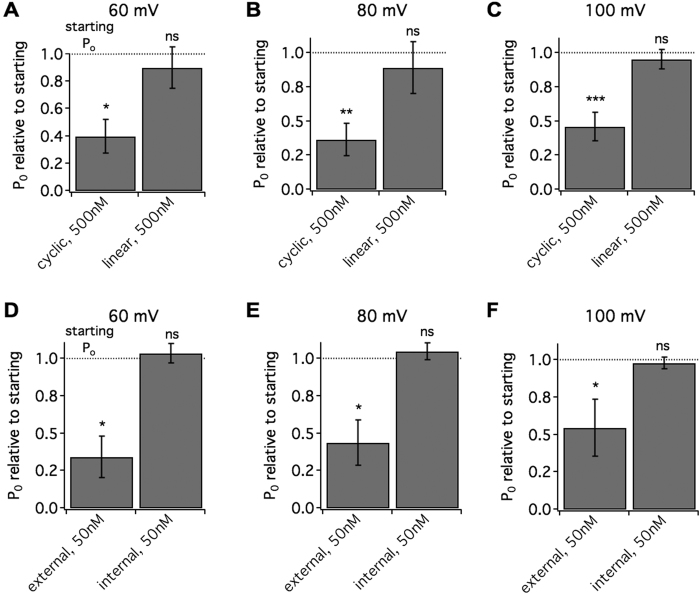
The cyclic form of LS3 accesses the human BK channels from the extracellular side to alter gating. Human BKα channels (ZERO isoform) expressed in HEK293 cells were recorded in inside-out patches. (**A**–**C**) Bar graphs displaying the post-peptide P_o_ relative to the starting P_o_ for either 500 nM LS3 (cyclic) or 500 nM of the reduced form of LS3 (linear). Unlike LS3, the linear form did not alter P_o_ (pre vs. post: *p < 0.05, **p < 0.01, ***p < 0.001, N = 6–12, planned Student’s t-tests). (**D**–**F**) Bar graphs displaying the post-peptide P_o_ relative to the starting P_o_ for 50 nM applied to either the external or internal side of the membrane. Only when applied to the external side did LS3 significantly reduce P_o_ at all voltages (pre vs. post: *p < 0.05, N = 6, planned Student’s t-tests).

Next we characterized how the peptide was interacting with the channel. To probe if the cyclic conformation of the peptide contributes to potency, we tested a linearized version of LS3 without the disulfide bridge and found it was less potent (Fig. 3A–C). At 500 nM, there was no significant reduction in P_o_ at any voltage. This suggests the disulfide bridge favors conformations that most readily interact with the channel. Constraint provided by the bridge may also increase binding stability^36^, contributing to picomolar activity. To gain insight into how the peptide accesses the binding site on the channel, LS3 was applied on either the extracellular or intracellular side of the channel. Application of 50 nM peptide to the extracellular side of the channel led to a reduction in P_o_ at each voltage (pre vs. post: p < 0.05, N = 6, Fig. 3D–F), while the same concentration of peptide on the internal side did not reduce P_o_ (pre vs. post: n.s., N = 6, Fig. 3D–F). These findings indicate that the peptide can readily access one or more binding sites that influence channel gating from the extracellular but not the intracellular side. It is unlikely then that the binding site(s) reside in the intracellular domain of the channel.

**Figure 4:**
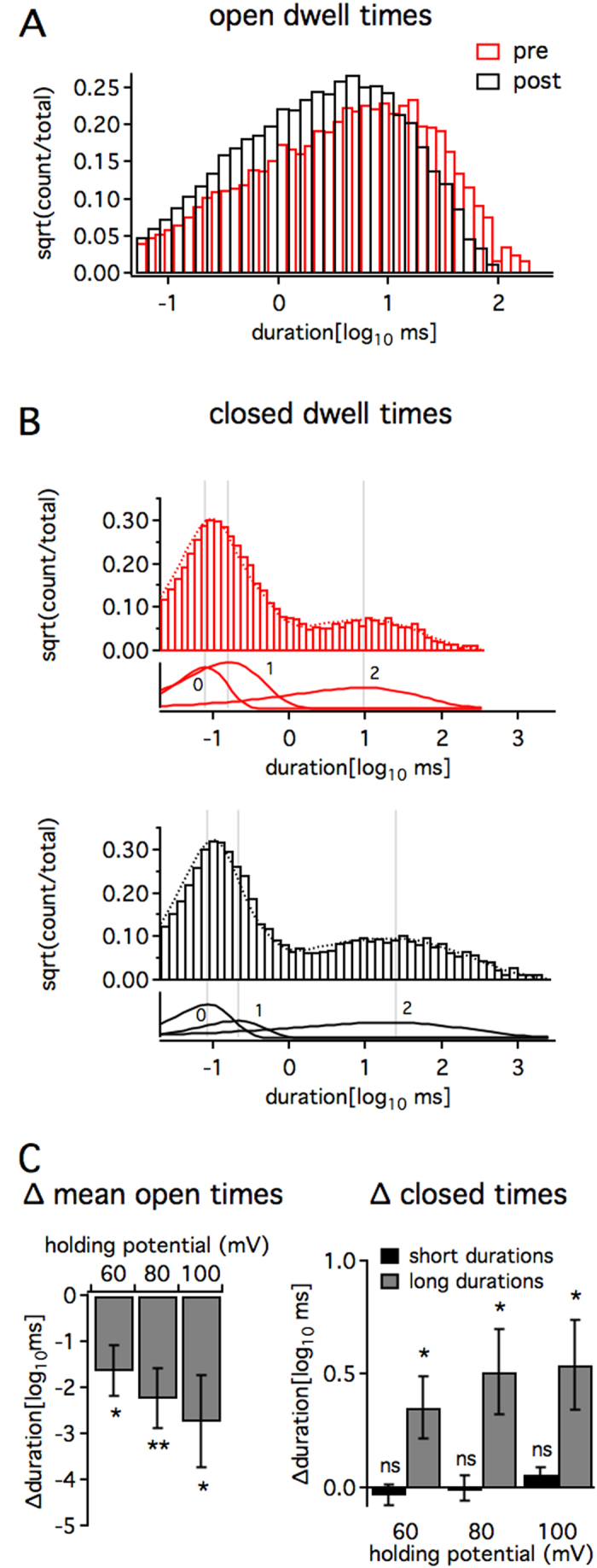
LS3 alters open and closed dwell times of the human BK channel. Single human BKα channels (ZERO isoform) expressed in HEK293 cells were recorded in inside-out patches. Intracellular calcium held at 750 nM. A-C, Bar graphs showing one example of changes in open and closed dwell times before (red) and after (black) 500 nM LS3 (100 mV). Channel open dwell times shortened (**A**). Three-component exponential fits of closed dwell times (**B**) showed that the longest duration dwell times (component 2) lengthened while short dwell times (component 0) did not change significantly. (**C**) Bar graph (right) of the change in mean open times (left) at each holding voltage shows that LS3 shortened openings (pre vs. post mean open times: *p < 0.05; N = 8, planned Student’s t-tests). Bar graph (left) of the change in peak times for short (black) and long (gray) duration closings shows that only the longest duration closings lengthened (pre vs. post peak times: *p < 0.05, **p < 0.01; N = 8, planned Student’s t-tests).

Our *in vitro* data demonstrates that LS3 reduces the probability of opening of the human BKα channel. To determine if LS3 does so by acting as a pore closer or blocker, we examined the change in open and closed times. The results show that LS3 alters human BKα channel gating by decreasing mean open times at all holding potentials (60–100 mV, p < 0.05, Fig. 4A,C). In contrast, long closed dwell times increased (p < 0.05, Fig. 4B,C), although short dwell times remained unchanged. The change in both mean open and closed times suggests that, rather than blocking the pore, LS3 modulates channel gating.

**Figure 5:**
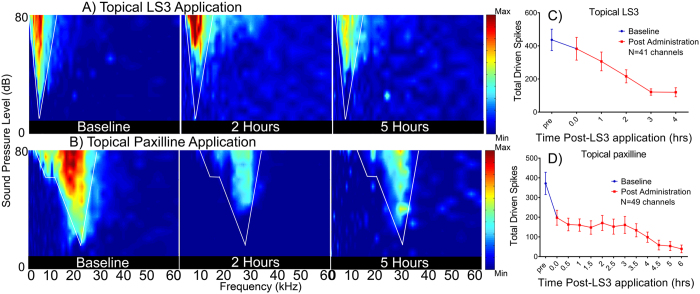
Topical application of LS3 suppresses sound evoked activity from the mouse auditory midbrain. Representative examples of two eFRAs from a young adult mouse are shown in the left most panel of (**A** and **B**) and were typically V-shaped with varying amounts of spontaneous activity. Total spike counts within the eRF, as denoted by the white lines, were measured before and after topical application of 1 μL of a 10 μM stock of LS3 (**A**) or paxilline (**B**) to the surface of the IC. Following topical application (panel A) a drop in sound evoked activity from 550 spikes, to 277 spikes at 2 hrs and stabilized at 300 spikes at 4 hrs. A similar effect was noted for paxilline where sound evoked driven activity decreased from 886 spikes to 392 spikes at 2 hrs. and 126 spikes at 5 hrs. (**B**). Mean data is shown in panel C from 47 recording sites and shows that after 3 hours driven mean sound evoked activity fell from 430 to 130 spikes, a ~70% reduction (N = 5 mice). Similarly, application of the pore blocker, paxilline (1 μL of 10 μM stock), to the surface of the IC (N = 5 mice) resulted in an 87% reduction in sound driven activity 5 hours post-application (**D**).

**Figure 6:**
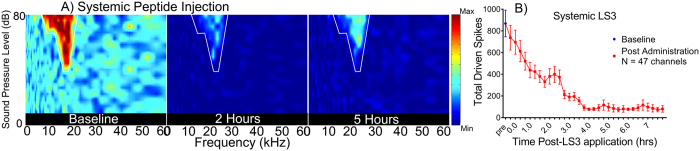
Systemic application of LS3 suppresses sound evoked activity from the mouse auditory midbrain. Example eRF having a BF of 19.5 kHz and a minimum threshold of 42 dB SPL. LS3 was administered via a systemic injection (0.3 mg/kg I.P.), and sound evoked activity was observed to decrease over a period of several hours from 522 spikes at baseline to 41 spikes at 2 hrs. and 37 spikes at 5 hrs. (**A**). Mean (±SEM) sound evoked activity from 47 units from 5 CBA mice in which LS3 was systemically administered. We observed a steady decline in sound evoked activity over the course of 2 hours, which accounted for an 85% reduction in spikes within the eRF (**B**).

Finally, to test for potential clinical applications in mammalian CNS, we determined if LS3 could alter *in vivo* sound evoked activity in the auditory midbrain of mice. BK currents are known to shape activity in the peripheral and central auditory system^12^,^37^,^38^, although their specific role in the inferior colliculus is not fully characterized. As such, the effects of LS3 were compared with a highly specific, small-molecule pan-BK channel pore blocker, paxilline^39^. Excitatory receptive fields were acquired from 49 multichannel recording sites confirmed to be within the IC. After baseline recording, we applied LS3 (1 μL of a 10 μM stock) or paxilline (1 μL of a 10 μM) topically to the surface of the dura and continuously monitored the eRFs from between 10–14 channels. Two representative eRFs are shown in Fig. 5(A,B). LS3 and paxilline reduced sound evoked driven activity within the RF by approximately 50% from the baseline pre-peptide condition. Mean (±SEM) data from 49 units show that driven activity within the receptive field declined from 450 spikes pre-peptide to ~225 spikes 120 minutes post application. Driven activity continued to decline out to 4 hours [F(5,206) = 5.085 P = 0.0002], (Fig. 5C). The effects of topical application of paxilline (1 μL of a 10 μM stock) were similar in 51 units, reducing maximum spike counts by 50% from 375 to 180 spikes within 30 minutes, with suppression of sound evoked activity increasing out to 6 hours [F(12,430) = 4.146 P < 0.0001], (Fig. 5D). We also tested the stability of recordings when only the DMSO vehicle was administered to the surface of the IC. eRF spike counts remained stable over the time course of acquisition in control experiments (Supp. Fig. 3). Thus, both LS3 and paxilline reduced sound evoked activity over a similar time course suggesting, (i) that LS3 has a similar action on driven activity in the IC as the highly specific BK channel blocker, paxilline and (ii) that, like paxilline, LS3 can cross the BBB as both were applied to the surface of the dura. Similar to topical application, when we administered LS3 systemically, sound evoked activity within the eRFs were reduced over a similar time course (Fig. 6). A representative eRF (Fig. 6A) shows that systemic administration of LS3 resulted in a 93% decrease in sound evoked activity with the receptive field over 5 hours with driven activity decreasing from 522 spikes to 37 spikes at 5 hours. Mean data from 49 units show that systemic LS3 reduced maximum spike counts from 400 to 200 within 90 minutes of the injection, and driven activity continued to decline out to 7 hours [(F(32,936) = 7.724 P < 0.0001], (Fig. 6B).

**Figure 7:**
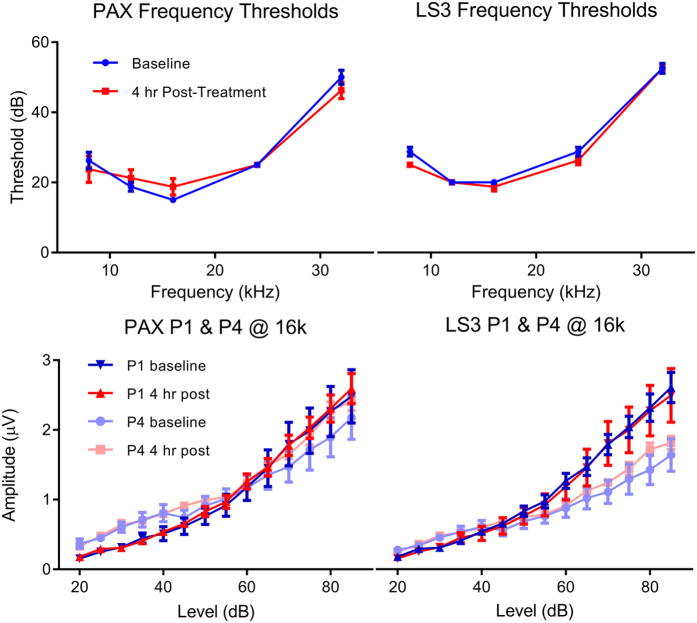
Systemic application of LS3 or paxilline does not alter hearing thresholds or suppress sound evoked activity of inputs to the inferior colliculus as measured with the auditory brainstem responses. Mean ABR thresholds (±SEM) at baseline (blue) and 4 hours (red) post I.P. injection of either paxilline (left) or LS3 (right). No significant effect of either BK channel modulator was observed. Mean amplitude by intensity functions for peak 1 (P1) and 4 (P4) of the ABR at baseline (blue symbols) and 4 hrs. following systemic injection (red symbols) of paxilline or LS3. Neither BK channel modulator reduced auditory nerve (P1) or brainstem (P4) amplitudes.

In order to rule out a loss of more caudal inputs to the IC we examined auditory brainstem responses (ABRs) to tone bursts prior to and following systemic injection of LS3. Peak 1 amplitude by intensity functions of the auditory nerve and generators caudal to the IC (peak 4) were unaffected by the BK channel modulators indicating that there is not a reduction in excitatory drive to the IC (Fig. 7). These results suggest that modulation of the BK channel within the IC is solely responsible for the change in sound evoked activity we observe following either topical or systemic administration of the peptide.

## Discussion

The large conductance calcium-activated potassium (BK) channel is a promising pharmacological target for many neurological applications. Here we characterize a novel BK channel-targeted peptide, LS3. Selected to have a compact scaffold with scalable synthesis, this novel peptide suppresses human BK channel gating *in vitro* with an IC_50_ in the mid-picomolar range. In addition to this high affinity activity at the BK channel, LS3 functional activity *in vivo* is highly specific. LS3 alters locomotion in both wild-type and BK channel humanized *Caenorhabditis elegans* via a BK channel-dependent mechanism. Experiments *in vivo* in mice showed that LS3 crosses the BBB and modulates sound evoked neural activity in the auditory midbrain. After either topical application on the dura or systemic administration, LS3 reduces sound evoked activity in the auditory midbrain *in vivo*. Auditory brainstem responses showed that, even with systemic injection, the LS3-induced modulation of sound evoked activity arises in the midbrain. The time-course of suppression of activity by LS3 for either application method matches that for a known small molecule BK channel pore blocker, paxilline. This suggests a similar mode of action for the two treatments and that, like paxilline, LS3 can cross the BBB. Together, our findings indicate that LS3 acts as a closer at BK channels in the auditory system to suppress sound evoked activity in the inferior colliculus. These findings support a number of studies demonstrating that BK-type channels within the IC play a crucial role in temporal processing and the ability to decipher complex sounds^37^,^38^,^40^.

CNS pharmacological applications favor pharmaceutical agents with high affinity, specificity, amenability to large-scale synthesis and BBB permeability. Peptide drugs have the potential for high affinity and specific binding to ion channel targets, as shown by voltage-gated potassium channel (Kv) peptide toxins. In addition to natural products, phage display screens have successfully selected peptides with high target affinity. For example, a phage display screen of scorpion toxin derivatives selected a novel peptide that blocks the Kv3.1 channel in the nanomolar range^41^. Here, a phage display screen was employed to select a high affinity BK channel targeted peptide. While fully randomized libraries tend to yield lower affinity interactions than derivative libraries, the monovalent display of the phage library employed for our screen supports the selection of interaction affinities in the pico to nanomolar range^42^. With an IC_50_ in the mid picomolar range, LS3 shows a higher apparent affinity than Kv peptide toxins or paxilline, a small molecule BK channel blocker with a low nanomolar IC_50_^39^. To probe the specificity of LS3, we employed the *C. elegans* behavioral assay because many gene products modulate *C. elegans* neuromuscular circuit activity^27^, including the highly conserved BK channel ortholog, SLO-1^28^,^29^. The finding that LS3 alters *C. elegans* locomotion in a BK channel-dependent manner indicates that LS3 acts specifically at the BK channel within an *in vivo* context. As *C. elegans* homologues have been identified for 60–80% of human genes including many highly conserved potassium channels^30^,^31^,^32^,^33^,^34^, this specificity of action for the BK channel likely carries over to mammals. Consistent with this idea, our findings show that LS3 does not have high affinity interactions with known ligand binding sites on 33 mammalian CNS targets.

Because of their complex structure, many peptides toxins are not suitable for large-scale synthesis. Forgoing a natural products scaffold, we employed a scaffold with scalable synthesis, a 9 amino acid peptide with an N- to C-terminal disulfide bridge. While the disulfide bridge adds an extra step in synthesis, it may be important for LS3 function. Constraint by the disulfide bridge restricts conformational change, potentially increasing binding stability with the target^36^. The disulfide bridge may contribute to the picomolar activity of LS3, as supported by the lack of effect of the linear peptide even in the high nanomolar range. Cyclic peptides also show more resistance to proteolytic degradation than linear peptides allowing them to survive longer application times^25^. Prolonged availability increases the amount that can cross the BBB^43^. However, the scaffold alone is unlikely to confer tissue permeability, since a systematic study has not supported the idea that cyclic peptides are more likely to be cell permeable^44^.

A pharmacological agent must cross the BBB in order to act at CNS-located targets. For LS3, 0.3 mg/kg I.P. suppressed sound evoked activity in the inferior colliculus without altering peripheral auditory processing. This functional activity arising in the CNS indicates that LS3 crosses the BBB. Many ion channel-targeted peptides are regarded as having low BBB permeability due to the large difference between the lethal dose when administered peripherally versus centrally^45^. For example, the LD_50_ for peripherally administered dendrotoxin is in the tens of mg/kg, while it is ten thousand fold lower with intraventricular injection^46^. In contrast, apamin, a small Kv toxin at 18 aa, has higher CNS availability^47^. A derivative of apamin can be detected in the brain after peripheral injection^48^. Interestingly, apamin is also known to alter learning and memory-related behaviors following intraperitoneal injection at a similar concentration used in our experiments^49^.

LS3, like other cell-penetrating peptides, may employ multiple methods to cross the BBB. One possibility is that LS3 membrane permeability is aided by arginines, which tend to cluster near the membrane surface and have a low energetic cost to partition into membrane^50^. LS3 contains only two arginines, fewer than typical cell-penetrating peptides, but conformation can influence the arginine requirement^51^. Rather than penetrating the membrane, LS3 may employ endocytosis at the BBB. This is the primary mode of transport for the arginine-rich, canonical cell and BBB-penetrating peptide, TAT. Finally, the ability to cross the BBB could be BK channel-specific. G protein-coupled receptors undergo peptide ligand-mediated internalization^52^, and this ligand can be modified to carry cargo across the membrane during receptor internalization^53^. BK channels are present in cerebral blood vessels^54^ and as such could be involved in BK channel-specific transport across the BBB.

Expressed throughout the peripheral and central auditory system^11^,^12^,^13^,^55^, the BK channel plays an important role in shaping auditory neuronal responses^12^,^13^,^40^,^55^,^56^. BK channel-mediated fast-hyperpolarization in the auditory system has been tied to processing of binaural cues involved in interaural phase modulation^57^,^58^. Within the IC, a key gateway for convergence of upstream and downstream auditory projections, BK channels modulate both excitatory and inhibitory inputs^40^. Spike timing is critical to neural processing of rapid sound features and altering BK channel function affects voltage regulation, and therefore discharge patterns, which are integral to transmitting synaptic information within the brain^40^,^59^. Our *in vitro* results indicate that LS3 significantly reduces BK channel gating, likely contributing to the reduced sound evoked firing *in vivo*. While BK channel activation provides a hyperpolarizing conductance, suppressing BK channel function reduces activity in some neurons due to the complex interaction of ionic conductances^10^,^12^,^60^. Together, these findings support a crucial role for BK-type channels within the IC in temporal processing and the ability to decipher complex sounds.

The BK channel has been implicated as regulating repetitive firing in neurons. Gated by both voltage and intracellular calcium, activation of BK channels hyperpolarizes neurons. Due to the complicated temporal interaction of varying ionic conductances, suppression of BK channel activity can reduce neuronal activity and vice versa. In the IC, suppressing BK current likely reduces the speed of repolarizing in these fast spiking neurons, slowing the rate of firing. Because the IC is a critical site for the initiation of audiogenic seizures, LS3 may be useful as an anti-convulsive. LS3 could also provide a potentially therapeutic action for tinnitus, which is associated with hyperexcitability in the central auditory system that is particularly apparent at the level of the IC and auditory cortex^17^,^18^,^19^,^61^.

LS3 suppresses sound evoked activity in the IC when administered systemically, but does not alter ABRs. Because cochlear function is altered by genetic^38^ manipulation of BK channel function, our findings suggest that LS3 is not active at cochlear BK channels. The presence of the BK β1 subunit in the cochlea but not IC could underlie this selectivity of action^62^,^63^,^64^. However, BK channels are also modified by splice variation, other accessory subunits and a number of post-translational modifications including phosphorylation and pamitoylation^65^,^66^. These other variations in channel composition could also lead to differential sensitivity and responses to BK channel manipulation by LS3 in the cochlea and IC^62^,^63^,^64^,^65^,^66^.

LS3 alters the function of BK channels across phyla, from nematode through human, *in vitro* and *in vivo*. Compared with existing high affinity peptide toxins, it is unique in its small size, simple structure, modification of channel gating and ability to cross the BBB. LS3 is not known to occur in nature. It also does not share sequence similarity with known BK channel binding partners, or, in cases where it has been studied, key interacting residues that confer peptide ligand activity^20^,^23^,^24^. Unlike many peptide toxins LS3 modulates gating, acting as a closer at the BK channel, rather than blocking the channel pore. For use *in vivo*, high affinity pharmacological agents that modify channel gating can be safer than pore blockers. As a BK channel closer, LS3 has potential therapeutic value as an anti-convulsive, particularly for audiogenic seizures, tinnitus or in treating certain cancers^5^,^6^,^9^,^10^. Future characterization of LS3 distribution in the brain and action at differently composed BK channels will pinpoint uses for underserved neurological applications.

## Methods

### Peptide selection and synthesis

A monovalent phagemid display library (library C, Mobitec) was sequentially panned against three sets of HEK293 cells (see below). For negative selection, cells expressed the human glycine receptor α1 (hGlyRα1, X52009) and the rat small conductance calcium-activated channel 2 (rSK2, U69882.1). For positive selection, cells expressed the human BKα channel ZERO isoform (NM_002238). The progress of the selection process was monitored after each panning round by titering and sequencing the phagemid DNA. With 6 randomized and 3 fixed amino acids, this library started with 3 × 10^7^ unique sequences. LS3 was selected as one of the sequences expressing motifs that were enriched more than 1000 -fold. LS3 was synthesized as a TFA salt at 98–99% purity verified by HPLC and MS analysis (Genscript, Piscataway, NJ). Stocks were dissolved in water at 10 mM and aliquots were lyophilized and stored at −80 °C. As a secondary determination of identity and purity, in-house LC/MS was performed on a single quadrupole Mass Spectrophotometer (Agilent 6130) interfaced with a HPLC with a diode-array (UV-vis) detector (Agilent 1200).

### *C. elegans* strains and transgenics

Worms were cultivated at 20 °C as described with OP50 bacteria^67^. Worms cultured on plates contaminated with fungi or other bacteria were excluded from this study. The reference wild-type strain was N2 Bristol. The reference *slo*-*1*(*null*) strain and background for transgenic strains was NM1968, harboring the previously characterized null allele, *js379*^29^. Multi-site gateway technology (Invitrogen) was used to construct plasmids. 2501 kb of the native *slo*-*1* promoter (*Pslo*-*1*) and the traditional *unc-54* UTR were used in combination with *slo*-*1a*(*cDNA*)::*mCherry* for a rescue construct. To test rescue with the human BK channel, an *hslo*(*ZERO isoform, cDNA*)::*mCherry* version was constructed. The *slo*-*1*(+) and *hslo*(+) plasmids were injected at a concentration of 20 and 10 ng/μl, respectively. The co-injection reporter PCFJ90 (1.25 ng/μl) was used to ensure proper transformation of the arrays. As such, JPS345 carried *vxEx345*, an extrachromosomal array containing [*Pslo*-*1*::*slo*-*1*::*mCherry*::*unc*-*54UTR*,*Pmyo*-*2*::*mCherry*::*unc*-*54UTR*]. JPS340 carried *vxEx345*, an extrachromosomal array containing [*Pslo*-*1*::*hslo*::*mCherry*::*unc*-*54UTR*,*Pmyo*-*2*::*mCherry*::*unc*-*54UTR*].

### *C. elegans* behavioral assays

Age-matched day one adults were cleaned of bacteria by letting them crawl around on an unseeded plate and then moved into a puddle of NGM or peptide dissolved in NGM on another unseeded plate. NGM and peptide treatment groups were always run in tandem to control for behavioral variance. While much shorter than typical drug applications in *C. elegans*^68^,^69^, brief application in liquid was chosen to avoid potential catabolic by-products of incubation on metabolically active *E. coli*. Higher LS3 concentrations were used to compensate. The puddle was refreshed 1–2 times as needed, but let to fully absorb into the agar by 30 minutes. After 30 minutes, crawl behavior was videoed (Flea2 camera, Point Grey Research, Canada; StreamPix 3, NorPix, Canada). Copper rings restricted movement to a proscribed area. The worms were tracked offline using custom macros (Image-Pro, MediaCybernetics, Rockville, MD) for 1 minute to obtain crawl speed (cm/min). Group means ± SEM for peptide-treated vs. vehicle-treated controls were compared at each concentration with Student’s t-tests. Rescue analysis was completed with two-way ANOVA (SigmaPlot, San Jose, CA). Crawl speeds for the peptide treated groups were also normalized to the performance of yoked controls. Normalized group means±SEM were compared vs. *slo*-*1* null performance by two-way ANOVA.

### HEK cell maintenance and transfection

HEK293 cells (ATCC, Manassas, VA) were grown according to standard procedures. Cells were cultured at 37 °C in a 5% CO_2_ atmosphere in Dulbecco’s modified Eagle’s medium with L-glutamine, sodium pyruvate and 10% fetal bovine serum (Invitrogen). Cell lines were split with trypsin/EDTA in Hanks’ balanced salt solution (Invitrogen) up to 25–30 cycles. For phage display, stable lines stably expressed rSK2 or hBKα ZERO isoform. Cells were transfected (Lipofectamine 2000, Invitrogen) with hGlyRα1 and used 48 hours later. For electrophysiological recordings, cells were transfected with hBKα ZERO isoform. Enhanced green fluorescent protein (EGFP) was cotransfected as a marker. Electrophysiological recordings were made 16–72 h after transfection. Although the profile of BK channel composition varies from tissue to tissue, the ZERO BK channel splice variant is widely expressed, particularly in the nervous system, serving as a representative form for studying the modulation of BK channel gating.

### Patch-clamp recordings

Voltage-clamp recordings were performed at room temperature (22–24 °C) using an inside-out configuration on patches pulled from HEK293 cells. The extracellular solution contained the following (in mM): 2 KCl, 136 KOH, 20 HEPES, 2 MgCl_2_, adjusted to pH 7.2 with MeSO_3_H. In order to apply peptide to the extracellular surface, patch electrodes (7–20 MΩ in resistance) were tip-filled with normal extracellular solution and backfilled with extracellular solution containing LS3. Enough normal extracellular solution was included to provide at least five minutes of peptide-free recording (determined by plotting P_o_ vs. time). The intracellular solution (in the bath) contained the following (in mM): 6 KCl, 132 KOH, 20 HEPES, adjusted to pH 7.2 with MeSO_3_H. To achieve 750 nM free Ca^2+^, 4.17 mL of 1 M CaCl_2_ and 5 mM EGTA were included, a ratio verified by measurement with a Ca^2+^-sensitive electrode. Voltage-clamp recordings made with an Axopatch 200 A amplifier and custom macros in IgorPro. Analysis was performed with QUB (http://www.qub.buffalo.edu), including P_o_, mean open time and three component exponential fits to closed dwell times. Group means±SEM for post-peptide measures were plotted relative to pre-peptide values and compared with pre values via planned paired t-tests.

### Receptor Binding Assay

LS3 was screened against a comprehensive panel of CNS-based proteins. Detailed protocols can be found within the US National Institute of Mental Health Psychoactive Drug Screening Program (NIMH PDSP) Assay Protocol Book (version II), by B. L. Roth, March 2013 (available at: http://pdsp.med.unc.edu/PDSP%20Protocols%20II%202013-03-28.pdf). Briefly, competition binding assays tested whether 10 microM LS3 significantly altered binding of known radioligands for 33 targets. Radioactivity in the presence of the LS3 (sample) was calculated with the following equation and expressed as a percent inhibition: % inhibition = (sample CPM – non-specific CPM)/Total CPM – non-specific CPM) × 100. Total binding was measured with no competing ligand. Non-specific binding was measured in the presence of reference compound. The % inhibition by LS3 was measured 4 times for each receptor. Less than 50% inhibition was considered insignificant as this suggests a Ki > 10 microM.

### Mouse subjects

Multi-channel recordings were acquired from young CBA/CaJ mice. CBA/CaJ mice were chosen because the loss of peripheral function is similar to humans, making them a good model for the study of presbycusis^70^,^71^,^72^,^73^. Founder breeding pairs were obtained from The Jackson Laboratory (Bar Harbor, ME), bred within the facilities of the university vivarium, and housed 3–4 per cage with litter-mates in rodent micro-isolator cages (36.9 × 15.6 × 13.2 cm) on a 12/12 hour light/dark cycle with ad lib water and food pellets. The temperature was maintained near 25 °C. Cages were changed weekly, and the mice were monitored for signs of distress several times throughout the day. Only nulliparous mice were used for experiments, while breeder mice were kept in separate cages. All procedures were preapproved by the University of South Florida Committee on Animal Resources and are consistent with US Federal and NIH guidelines under IACUC protocol #0245 R.

### Surgical preparation

The mice were initially anesthetized with an intraperitoneal (I.P.) injection of ketamine and xylazine (100 mg/kg and 10 mg/kg). After anesthesia was induced, the top of the animal’s head and neck was then shaved of fur to prevent contamination of the incision site. The skin was cleaned with germicidal scrub, rinsed with 70% alcohol, and prepped with iodine. The skull was then exposed, 2% lidocaine was applied to the site of incision, and a small brass tube was secured to the skull surface along the sagittal suture at bregma with vet bond and adhered with dental cement. Mice were given a recovery period of 24–48 hours before beginning the experimental sessions.

### Drug administration

The peptide was prepared from a 10 mM aqueous stock solution and diluted down to 10 μM. Topical administration of either peptide or paxilline consisted of direct application of 1 μL solution to the exposed surface of the inferior colliculus at concentration dosages of 10 μM or an I.P. injection at a dose of 10 μg, which is 0.33 ng/mg body weight for a 30 g mouse. Fresh solutions were made prior to each experiment.

### Auditory Brainstem Response Procedures

ABR recordings were acquired after the mice were anesthetized with ketamine (120 mg/kg) and xylazine (10 mg/kg) I.P., and respiration was monitored throughout to determine when additional supplemental doses were needed. Body temperature was kept constant at 37 °C using a feedback controlled heating pad (Physitemp TCAT2-LV Controller, Clifton, NJ). Stimuli and recordings were generated digitally and controlled using a TDT RZ6 Multi-I/O Processor and their BioSig/SigGen software. Acoustic signals were played through a multi-field (MF1) magnetic speaker (TDT, Alachua, FL) with a total harmonic distortion < = 1% from 1 kHz to 50 kHz, centered 0° azimuth in regards to the animal at a distance of 10 cm from the ear pinna. Tone bursts were presented at frequencies of 6, 12, 16, 20, 24, and 36 kHz (3 ms duration, 1 ms rise/fall time, alternating polarity) at a rate of 29 per second, attenuated in 5 dB steps from 80 dB SPL to 15 dB below threshold or 5 dB SPL, whichever was lower. Threshold was determined by visual inspection as the lowest intensity level which produced a defined wave in both replicates. All signals were calibrated using a Larsen Davis preamplifier, model 2221, with a 1/4″ microphone and a Larson Davis CAL200 Precision Acoustic Calibrator (PCB Piezotronics, Inc., Depew, NY). ABR recordings were acquired using a TDT RA4LI low-impedance digital headstage and RA4PA Medusa preamp with the active (noninverting) electrode inserted at the vertex, the reference (inverting) electrode below the left ear, and the ground electrode below the right ear. The responses were amplified (20×), filtered (300 Hz–3 kHz), and averaged using BioSig software and the System III hardware (TDT) data-acquisition system. A total of 256 tone burst signal and 150 GIN signal recordings were replicated for each acquisition, and muscle artifacts exceeding 7 μV were rejected from the averaged response. All recordings took place in a soundproof booth lined with echo-attenuating acoustic foam. ABR waveforms were analyzed using a custom MatLab program that automatically determined peak latencies and amplitudes in combination with secondary verification by an experimenter blind to the treatment group.

### Extracellular Recording Procedures

The right IC was stereotaxically located^74^ and exposed via a small (<1.0 mm) craniotomy. Prior to recording, chlorprothixene (Taractin®, 5–12 μl/g i.m.) was administered. The animal was then secured in a custom stereotaxic frame (Newport-Klinger) that was located in a heated (34 °C) chamber lined with sound-absorbing foam (Sonex®). Multi-unit extracellular activity was recorded using vertically oriented single shank silicon acute penetrating 16-channel electrodes with an impedance ranging from 1.2 to 2.1 MΩ (Type-A, 3 mm × 100 μm; NeuroNexus Technologies). The electrode was positioned stereotaxically over the IC after reference to the lambda landmark on the skull, and was advanced dorsoventrally into the IC by a micro positioner (Newport-Klinger PMC 100). The output from the electrode was attached to a low noise (5–6 μV noise floor) pre-amplifier (RA16), having an operating range of ±7 mV. Neural events were acquired and visualized in real-time using the OpenEx software platform (TDT, Inc.) and a custom designed MATLAB® (The MathWorks, Inc., Matick, MA) graphical interface. Neural recordings from each channel were then filtered (300–3000 Hz), amplified, and sampled at 25 kHz in a 1.25 ms time window subsequent to the event crossing a voltage discriminator. A spike triggering threshold of 4:1 signal to noise ratio (SNR) was automatically set for all channels. The search signal used to estimate the spike triggering thresholds was a 50-ms broadband noise stimulus presented at 60 dB SPL at a rate of 5/s. Each penetration typically yielded 8–16 active channels. Recording sessions lasted an average of 6–8 hours, and if at any time a mouse showed signs of discomfort, like excessive movement, it was removed from the apparatus and testing was halted.

### Stimulus generation and presentation

Noise and tone bursts were generated digitally (Real-time Processor Visual Design Studio (RVPds), TDT) using a System 3 processor and D/A converter (TDT RX6) with 200 kHz sampling rate. The signals were routed to an electrostatic speaker (TDT ES1) with a flat frequency response from 4 to 110 kHz. This speaker was placed at 60° azimuth contralateral to the recording site. Harmonic distortions were measured with a Dynamic Signal Analyzer (HP 35665 A) and were at least 60 dB below the primary signal. The distance between the speaker and the pinna was fixed at 22.5 cm and calibrated using a B&K 2610 amplifier and a 1/4″ microphone placed at the location of the pinna. eRFs from all active channels were acquired simultaneously using 25 ms (5 ms rise/fall) tone burst stimuli presented from 0 to 90 dB SPL in 5 dB steps and from 2 to 64 kHz for a total of 2125 frequency and intensity combinations that were presented pseudo-randomly five times at a rate of 10/s.

### Spike sorting

Spike waveforms were processed in MATLAB® using the TDT OpenDeveloper ActiveX controls and passed to AutoClass C v3.3.4, an unsupervised Bayesian classification system that seeks a maximum posterior probability classification, developed at the NASA Ames Research Center^75^,^76^. AutoClass scans the dataset of voltage–time waveforms according to custom specified spike parameters to produce the best fit classifications of the data, which may include distinct single- and multi-unit events, as well as noise. To discriminate the signal from noise in the present study, the variance of the background noise was estimated as the quartile range of the first five digitization points of the spike waveform, as these are recorded prior to the threshold-crossing event. To avoid overloading AutoClass with excessive noise, which leads to over-classification, this noise measure is used to screen the event waveform data, such that only voltage points with absolute values greater than this noise floor were presented for use in the classification. Once the classes had been determined in each channel of data, they were visualized within a custom MATLAB® program and assigned to multi-unit, single-unit, or noise classes. Event classes which were categorized as noise were subsequently discarded, and units with distinct biphasic waveforms and good SNR were classified as single-units. As most channels recorded information elicited from the spiking of two or more neurons, all recordings units in this paper were considered to be MUA^77^. Nonetheless, there was no observation of any consistent differences in the RFs between single units and multi-unit clusters.

### Data analyses

FRAs were analyzed using a custom MATLAB® program We classified RF tuning using a method similar to that used to classify neurons in the primary AC^78^. The frequency at which driven activity is responsive at the lowest intensity (threshold) is classified as the characteristic frequency (CF) and the point in the receptive field, which elicits the maximal driven activity is categorized as the best frequency (BF). A custom MATLAB® program was used to calculate the edges of each eRF, and this was verified via visual inspection to ensure no non-driven activity was included in the calculation. The edges of the RF were defined, in 10 dB steps above threshold, as the activity levels that were equal to or greater than the background rate and at least 15% of the maximum rate. Each RF was categorized into low-BF (<15 kHz), mid-BF (15–30 kHz), and high-BF (>30 kHz) groups based on the topographical representation proposed by Willott^79^. The maximum driven rate, as well as the total spike counts, were taken from the baseline.

Statistical analysis and graphs were created using GraphPad Prism version 6.01 for Windows (GraphPad Software, La Jolla, CA). The majority of the data results are presented using box plots, which allows for both mean, along with the ± standard error of the mean (SEM) and median values to be denoted. A one-way ANOVA test and Tukey’s repeated measures analysis procedure were used to evaluate the effects of the peptide on the spike counts within the baseline eRF. Alpha was set at 0.05 for all statistical tests.

## Additional Information

**How to cite this article:** Scott, L. L. *et al*. A novel BK channel-targeted peptide suppresses sound evoked activity in the mouse inferior colliculus. *Sci. Rep.* **7**, 42433; doi: 10.1038/srep42433 (2017).

**Publisher's note:** Springer Nature remains neutral with regard to jurisdictional claims in published maps and institutional affiliations.

## Supplementary Material

Supplemental Figures and Table
