# Supplementary material for: A novel BK channel-targeted peptide suppresses sound evoked activity in the mouse inferior colliculus
Source: Sci Rep. 2017 Feb 14;7:42433. doi: 10.1038/srep42433 (PMC5307958; doi:10.1038/srep42433)
Supplement: Supplemental Figures and Table [file srep42433-s1.pdf]

Supplemental figures and table

Title: A novel BK channel-targeted peptide suppresses sound evoked activity in the mouse inferior colliculus

Authors and affiliations:

L.L. Scott[1*], E.J. Brecht[3], A. Philpo[1], S. Iyer [1], N.S. Wu[1], S.J. Mihic[1], R.W. Aldrich[2], J. Pierce[1,2], J.P. Walton[3]

1. Waggoner Center for Alcohol and Addiction Research, University of Texas at Austin

2. Center for Learning and Memory, University of Texas at Austin

3. University of South Florida

*corresponding author:

The University of Texas at Austin, Department of Neuroscience

2506 Speedway C7350

NMS5.234

Austin, TX 78712

512-507-0970

luisa_scott@utexas.edu

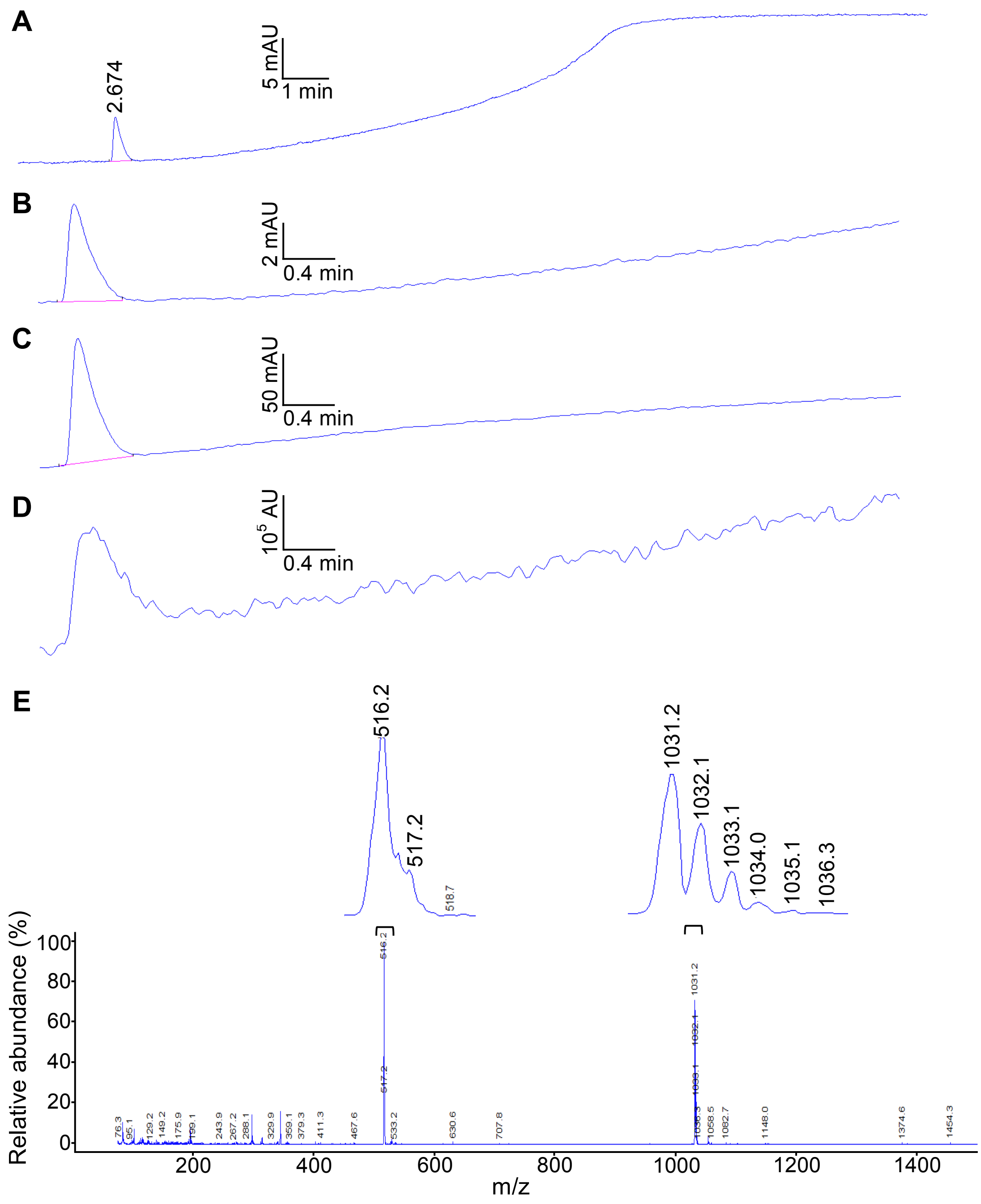

Supplemental Figure 1. LC/MS confirmed purity and identity of LS3. A-C, HPLC traces showed a single peak absorbance at 2.674 min for both 254 nm (A, 1-20.5 min; B, 2.4-9 min) and 214 nm (C, 2.4-9 min). D, The MS total ion current trace also showed a single peak (2.4-9 min). E, The mass spectrum indicates a molecular weight of ~1032 g/mol, similar to the predicted mass (1033.28 g/mol) for the peptide.

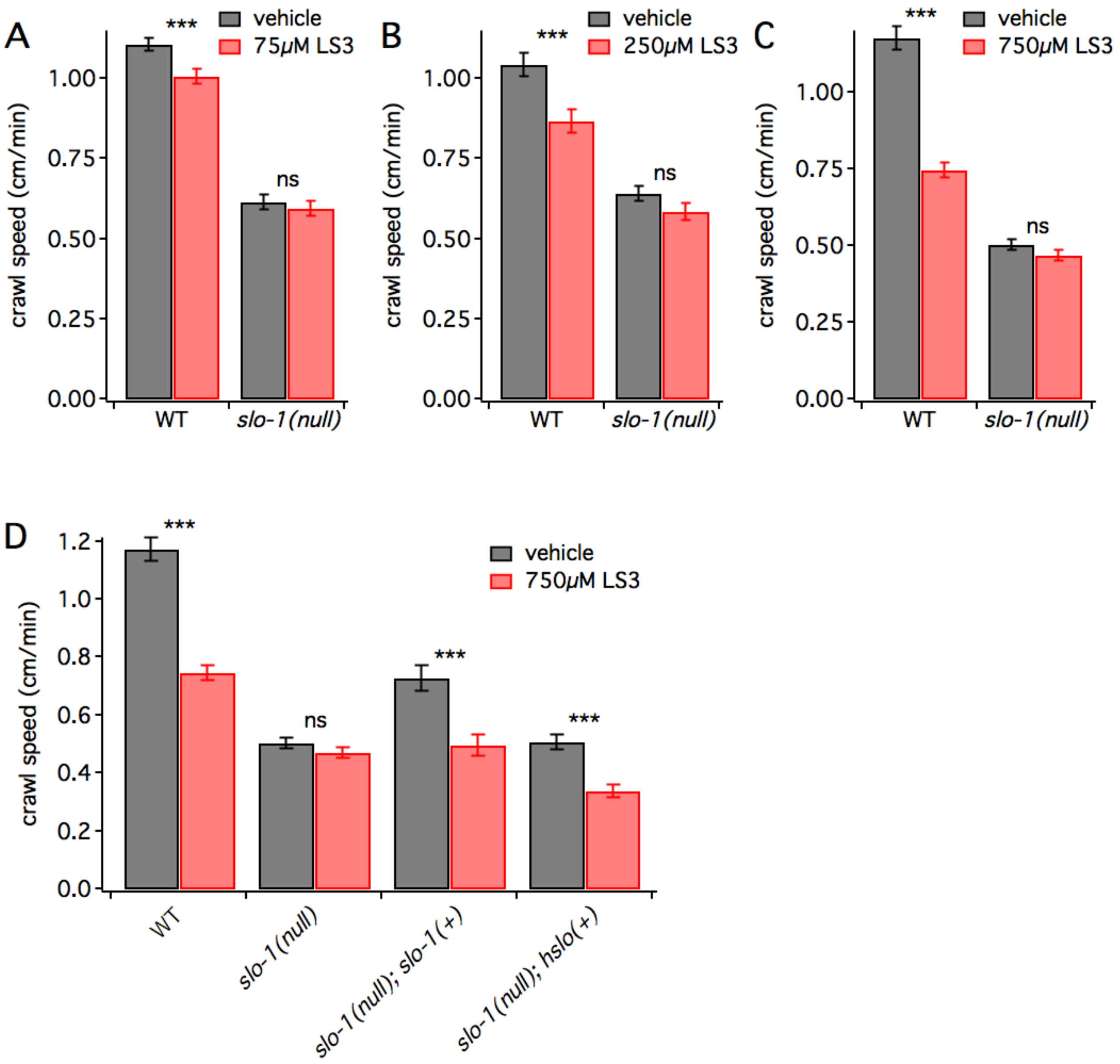

Supplemental Figure 2. LS3 alters BK channel function in wild type or humanized *C. elegans*. A-C, Bar graphs of crawl speeds for vehicle- (gray) and LS3-(red) treated groups show that LS3 reduced crawl speed for wild type but not *slo-1(null)* worms at 75 µM (A), 250 µM (B) and 750 µM (C; LS3 vs. vehicle for all concentrations: ***p<0.001, N=81-210, planned Student's t-tests). D, Bar graph of crawl speeds shows that a reduction in crawl speed by 750 µM LS3 was rescued on the *slo-1* null background with extrachromosomal expression of

either the worm (*slo-1(+)*) or the human (*hslo(+)*) BK channel gene (LS3 vs. vehicle: ***$p<0.001$, N=78-162, planned Student's t-tests).

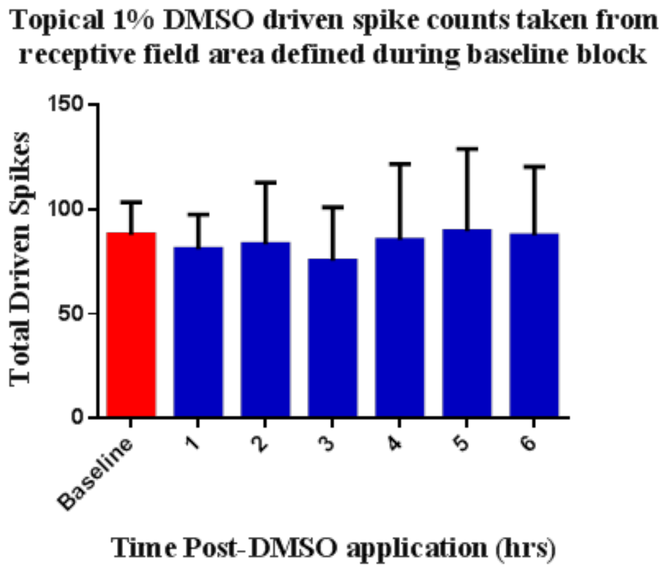

Supplemental Figure 3. Topical application of 1uL of 1% DMSO does not alter sound evoked activity from the mouse auditory midbrain. Total spike counts (for 9 units) within the eRF were measured before and up to 6 hours after topical application of DMSO. eFRAs were measured at 1 hour intervals and even after 6 hours, driven activity remained stable (N=1).

Target	Radioligand	% inhibition by 10 μM LS3
Ion channels and receptors		
HERG	Dofetilide	21.2
$GABA_A$	Muscimol	19.3
Peripheral benzodiazepine	PK11195	-7.6
5HT 1A	8-OH-DPAT	11.4
5HT 1B	GR127543	2.8
5HT 2A	Ketanserin	16.2
5HT 2B	LSD	3.2
5HT 3	LY278574	-10.3
5HT 5A	LSD	-3.2
5HT 6	LSD	-0.7
5HT 7	LSD	-1.6
Dopamine D1	SCH233930	15.7
Dopamine D2	N-methylspiperone	20.9
Adrenergic Alpha1A	Prazosin	11.3
Adrenergic Alpha2A	Clonidine	3.2
Adrenergic Beta2	Iodopindolol	-12.7
mAcetylcholine M1	QNB	12.6
mAcetylcholine M2	QNB	-9.6
mAcetylcholine M3	QNB	11.5
mAcetylcholine M4	QNB	6.8
mAcetylcholine M5	QNB	-5
Histamine H1	Pyrilamine	14.8
Histamine H2	Tiotidine	-23.1
Histamine H3	α-methylhistamine	5.1
Opioid DOR	DADLE	1.9
Opioid KOR	U69593	-7.7
Opioid MOR	DAMGO	2.2
Sigma 1	Pentazocine	-9.4
Sigma 2	DTG	24.3
BZP rat brain site	Flunitrazepam	5.1
Transporters		
DAT	WIN35428	-8.2
NET	Nisoxetine	6.7
SERT	Citalopram	0.1

Supplemental Table 1
